# Setting up a clinical trial in care homes: challenges encountered and recommendations for future research practice

**DOI:** 10.1186/1745-6215-16-S2-P198

**Published:** 2015-11-16

**Authors:** Victoria Shepherd, Jacqui Nuttall, Kerenza Hood, Nadine Aawar, Christopher Butler

**Affiliations:** 1 Cardiff University, Cardiff, UK; 2 Oxford University, Oxford, UK

## Background

Older adults in care homes have increasingly complex health care needs, however there is currently a lack of evidence for much of the care provided for this population. Recruitment of frail, older people to research is a complex process and often results in care home residents being excluded from research participation. This presentation draws on the experience of setting up a randomised controlled trial to determine the effectiveness of probiotics on antibiotic-associated diarrhoea in care home residents (Probiotics for Antibiotic Associated Diarrhoea in Care Homes (PAAD) Study).

## Findings

Significant challenges were encountered setting up a clinical trial in care homes that were unique to this research setting. Challenges included ethics committee concerns regarding the inclusion of adults lacking capacity, indemnity, and non-NHS research governance. The classification of the study intervention (a widely available food supplement with a low risk safety profile) as an investigational medicinal product, with the associated requirements including obtaining statutory approvals and research governance, had a major impact.

## Conclusion

The process for setting up a clinical trial in care homes has been more complex and time consuming than the process for setting up an observational study in the same setting, and clinical trials in other health care settings. We recommend regulatory changes to ensure approvals processes are more proportionate to risk and context, to ensure that care home residents have the opportunity to participate in research and are able to help generate much needed evidence to underpin care. Recommendations made may inform future research practice.

